# Scalable amplification of strand subsets from chip-synthesized oligonucleotide libraries

**DOI:** 10.1038/ncomms9634

**Published:** 2015-11-16

**Authors:** Thorsten L. Schmidt, Brian J. Beliveau, Yavuz O. Uca, Mark Theilmann, Felipe Da Cruz, Chao-Ting Wu, William M. Shih

**Affiliations:** 1 Department of Cancer Biology, Dana-Farber Cancer Institute, 450 Brookline Avenue, Boston, Massachusetts 02215, USA; 2 Department of Biological Chemistry and Molecular Pharmacology, Harvard Medical School, 240 Longwood Avenue, Boston, Massachusetts 02115, USA; 3 Wyss Institute for Biologically Inspired Engineering at Harvard, 3 Blackfan Circle, Boston, Massachusetts 02115, USA; 4 Center for Advancing Electronics Dresden (cfaed), Technische Universität Dresden, 01062, Dresden, Germany; 5 Department of Genetics, Harvard Medical School, 77 Avenue Louis Pasteur, Boston, Massachusetts 02115, USA

## Abstract

Synthetic oligonucleotides are the main cost factor for studies in DNA nanotechnology, genetics and synthetic biology, which all require thousands of these at high quality. Inexpensive chip-synthesized oligonucleotide libraries can contain hundreds of thousands of distinct sequences, however only at sub-femtomole quantities per strand. Here we present a selective oligonucleotide amplification method, based on three rounds of rolling-circle amplification, that produces nanomole amounts of single-stranded oligonucleotides per millilitre reaction. In a multistep one-pot procedure, subsets of hundreds or thousands of single-stranded DNAs with different lengths can selectively be amplified and purified together. These oligonucleotides are used to fold several DNA nanostructures and as primary fluorescence *in situ* hybridization probes. The amplification cost is lower than other reported methods (typically around US$ 20 per nanomole total oligonucleotides produced) and is dominated by the use of commercial enzymes.

## Abstract (web summary)

Synthetic oligonucleotides are the main cost factor for studies in DNA nanotechnology. Here, the authors present a selective oligonucleotide amplification method, based on three rounds of rolling-circle amplification, that produces nanomole amounts of single-stranded oligonucleotides per millilitre reaction.

## Graphical abstract

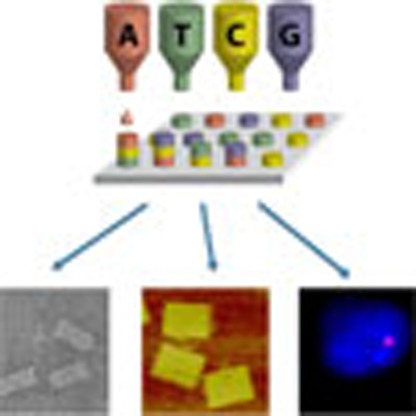

Many applications in DNA nanotechnology, genetics, synthetic biology and proteomics require thousands of single-stranded oligonucleotides. The price for standard column-synthesized oligonucleotides had steadily decreased in the last decades but has stabilized at around US$ 0.10 per base and can therefore still dominate the cost of certain studies or make them prohibitively expensive. Examples for such application include DNA origami^1^,^2^,^3^; single-stranded tile^4^ or brick structures^5^; multiplexed PCR and targeted sequencing^6^; gene and genome synthesis^7^,^8^,^9^,^10^; multiplexed genome engineering^11^,^12^; and fluorescence *in situ* hybridization (FISH)^13^,^14^,^15^.

Microarray-chip-synthesized oligonucleotide libraries can be produced by inkjet printing^16^,^17^, electrochemical or light-assisted synthesis methods^18^. The commercial price per base is around two orders of magnitudes lower than for column-synthesized oligonucleotides and has the potential to drop with increasing demand, as the reagent cost does not dominate the production. Such oligonucleotide libraries can contain a few thousands to hundreds of thousands of distinct programmable oligonucleotide sequences. A limitation of all microarray synthesis methods is that only few attomoles (10^−18 ^moles) per oligonucleotide sequence are produced. Although most applications do not require the tens of nanomoles provided by conventional column-based oligonucleotide synthesis, sub-femtomole amounts often do not suffice, either.

A number of enzymatic oligonucleotide amplification methods have been employed to obtain larger quantities of oligonucleotides from libraries. The most popular amplification method, the polymerase chain reaction (PCR), has also been successfully employed^6^,^7^,^8^,^15^,^19^,^20^; however, it has major drawbacks. First of all, double-stranded DNA is produced, whereas for many applications, including structural DNA nanotechnology, single-stranded DNA is required. Therefore, single-stranded fragments have to be recovered by labour-intensive and lossy denaturing gel electrophoresis or with expensive magnetic beads. Second, PCR is not easily scalable; for example, a 96-well plate of one reaction yields only 2 nanomoles (that is, tens of micrograms)^15^. This, furthermore, limits multiplexing and automation of oligonucleotide amplification using PCR. Strand displacement amplification could be an interesting alternative to PCR as oligonucleotides are directly produced, but so far has been limited to nanogram amounts^21^ or a fourfold amplification after chip synthesis^22^. Another method was recently presented to amplify virtually error-free oligonucleotides with exactly controlled stoichiometry^23^. This method involves the synthesis of a sequence-verified pseudogene containing a few of the desired oligonucleotides, a cloning step into and amplification by *Escherichia coli* cells, sequencing of colonies and so on. This method is labour-intensive and expensive and therefore is limited to the production of only a few oligonucleotides at a time, and is mainly of interest for applications where the quality and stoichiometric ratios of oligonucleotides is crucial. However, many applications such as DNA origami or FISH do not require tight stoichiometric control and can tolerate an error rate of the oligonucleotide sequences similar to that of unpurified synthetic oligonucleotides.

Here we present a scalable one-pot *in vitro* method that is suitable for the parallel production of thousands of oligonucleotides based on circle-to-circle amplification (c2ca)^24^. In their manuscript, Dahl *et al.* described a billionfold amplification of ssDNA (to microgram amounts) after three sequential rounds of rolling-circle amplification (RCA) and showed that the method results in a largely reduced sequence-dependent amplification bias compared with PCR^24^. Two challenges for preparative amplification from microarray-derived pools are that amplified sequences retained fixed sequence domains at their two ends, and that no method was provided for selective amplification of subpools from the larger pool. We describe how these two challenges can be overcome for the parallel production and purification of subsets of hundreds or thousands of monomeric oligonucleotides from larger barcoded oligonucleotide libraries. These oligonucleotides can have practically arbitrary sequences, as fixed sequence domains are cleaved off using nicking endonucleases. Our process is more economical than any of the previously published amplification methods.

## Results

### Barcoded c2ca

**Figure 1:**
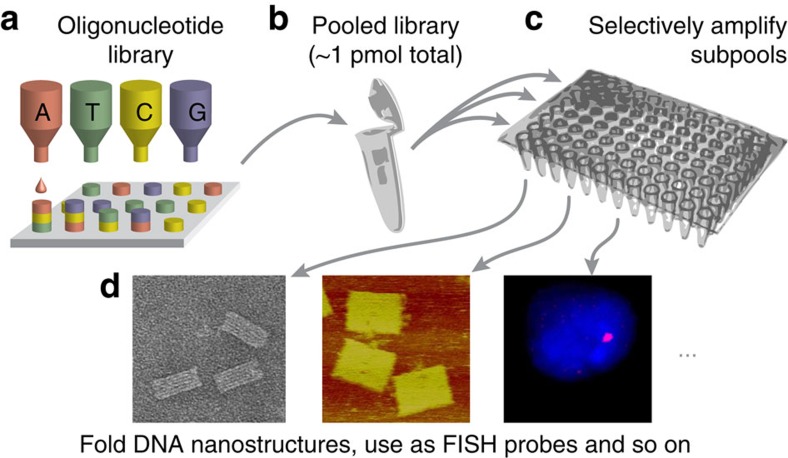
Schematic workflow of oligonucleotide amplification from oligonucleotide libraries. (**a**) Production of an oligonucleotide library. (**b**) All strands of the library combined typically add to only few picomoles. (**c**) Aliquots of the library are selectively amplified by circle-to-circle amplification up to a billionfold. An amplified subpool only contains a subset of oligonucleotides that is required for a certain experiment such as (**d**) the folding of a specific DNA nanostructure or to hybridize to a chromosomal target region for fluorescence *in situ* hybridization imaging.

For our method, we use an oligonucleotide library as a template that can be produced on a microchip either by inkjet printing^16^,^17^ (as drawn in Fig. 1a) or by other techniques^18^. Such a library can contain up to one million distinct sequences and is cleaved off the solid support (Fig. 1b). Small aliquots of the entire library are used as the template for the amplification of subsets of strands (‘subpools'; Fig. 1c). The three sequential rounds of RCAs are performed on each aliquot in parallel (details below), for example, in 96-well plates, where each well corresponds to one subpool. A subpool can, for example, contain ∼150–200 different oligonucleotides required to fold one DNA origami structure or can comprise several hundred or thousand primary FISH probes for a specific genomic target site (explanations below). The amplification reaction can be performed as a one-pot reaction, and only one final workup step is required for purification.

**Figure 2:**
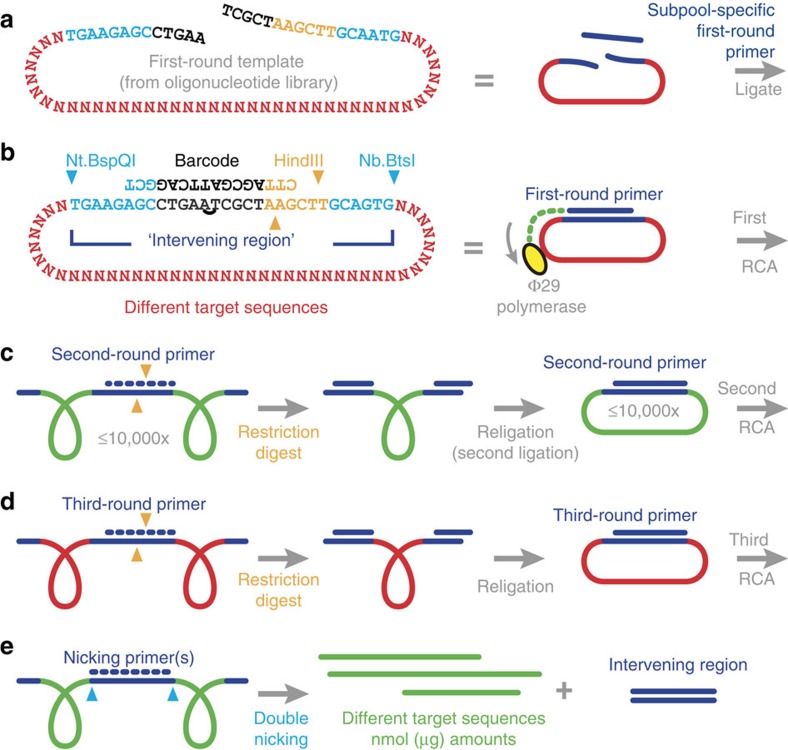
Oligonucleotide amplification by circle-to-circle amplification. (**a**) Subpool-specific first-round primers determine which subpool is amplified in the respective well of the 96-well plate (as in Fig. 1c). Primers are orthogonal and complementary to only one subpool-specific barcode set of the library and parts of the common sequence (details in Supplementary Note 2) and therefore only hybridize to the strands of the respective subpool and do not bind to template strands of other subpools. The targeted template strands are cyclized via ligation into circular template strands. (**b**) A polymerase with high processivity and strand displacement capacity is added (Phi29, yellow). Initiating at the first-round primer, the polymerase synthesizes concatenated (chain-like) repeated copies of the circular template (green is complementary to red). Non-circularized template strands of other subpools are not amplified. Under optimal conditions, we observed up to 10,000 concatenated copies of each template molecule for one RCA (quantitative real-time PCR experiments, data not shown). However, this amplification rate by itself is not sufficient for most applications, as each library contains only attomoles (10^−18 ^moles) per oligonucleotide sequence and only a small aliquot (for example, 1/1,000) of the library is used as a template for each amplification. (**c**) A second-round primer (blue dotted line) is hybridized to the intervening region of the first concatemer and the resulting double-stranded recognition site (orange) is digested with a restriction enzyme (HindIII) into monomers. A heat-inactivation step is necessary to inactivate the restriction enzyme for the next steps and is performed after most of the enzymatic reactions (details in Methods). The cut fragments of the second-round primer dissociate during this step. On cooling, an excess of the second-round primer hybridizes to the cut monomeric units and colocalizes the ends for a second ligation and RCA step. This second circular template has the reverse complementary sequence of the first circular template. (**d**) A third-round primer is annealed to the second concatemer (red again). Restriction digest, re-ligation and RCA are repeated to yield the final concatemer (**e**). One or two nicking primers are hybridized to this concatemer. A double-nicking reaction excises the intervening region from the target sequences (green). The nicking enzymes cut outside of their recognition sites; therefore, the entire intervening sequence can be removed to yield the (green) sequences of interest of a given subpool. Until this point, the entire process can be performed as a one-pot reaction without any intermediary workup steps. The excess of nicking primers, the intervening sequences, residual undigested concatemers and the excess of primers and enzymes can be removed using a final anion exchange chromatography step (see Methods).

To enable the selective amplification of oligonucleotides, we extended the reverse complement of each target sequence (red poly-N sequences in Fig. 2a) with two nicking sites (blue), a restriction site (orange) and one or two orthogonal subpool-specific barcodes of 10 nucleotides (nt) each (black; detailed discussion of barcode designs in Supplementary Note 1, Supplementary Fig. 1 and sequences in Supplementary Note 2). Whereas the nicking and restriction sites are shared by all strands of the library, barcodes are subpool-specific. The ‘intervening region' (dark blue) comprises all non-target sequences and is removed after the final amplification (Fig. 2e).

**Figure 3:**
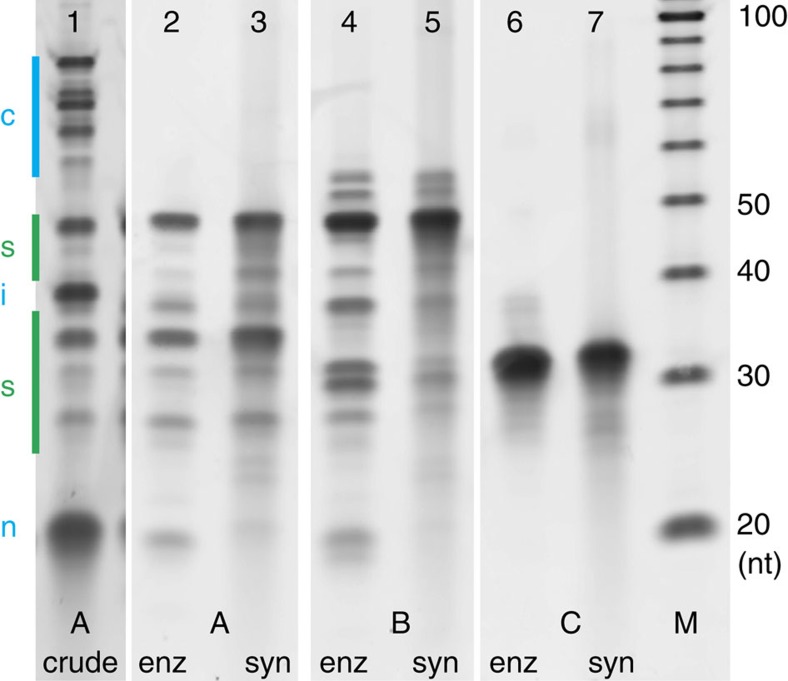
Oligonucleotide quality. PAGE analysis of three different subpools (A, B and C) synthesized by enzymes (enz) or else chemically (syn). Lane 1: crude product of final double-nicking reaction. Bands marked in green are staple strands, bands marked in blue are undesired concatemers (c), the intervening sequence (i) and the excess of two nicking primers (n) and can be removed well using HPLC purification (lane 2). Lanes 2, 4 and 6 are HPLC-purified c2ca reactions. Lanes 3, 5 and 7 contain unpurified synthetic oligonucleotides from a commercial vendor. M is a size marker.

As in the original report of c2ca^24^, we achieved about a billionfold amplification from three rounds. We detected no crosstalk between subpools by denaturing polyacrylamide gel electrophoresis (PAGE). Only gel bands of oligonucleotides present in the respective subpool could be seen; different lengths present only in other subpools were never observed. Control experiments with wrong or no primer sequences showed no detectable amplification. Lane 1 in Fig. 3 shows a typical result after the final double-nicking step. Incompletely cut concatemers (**c**) need to be removed for many applications, for example, to prevent multimerization or misfolding of structures in DNA origami. For this, preparative denaturing PAGE would be the standard method but is a very labour-intensive and lossy process, especially when products are of different lengths. Instead, we developed an automatable anion exchange high-performance liquid chromatography (HPLC) purification strategy (details in Methods). We recovered mixed length oligonucleotides with length between 25 and 55 nt for folding DNA nanostructures (Fig. 3, lane 1 before purification, lane 2 after purification) or 70-mers for FISH studies, with only traces of nicking primers and intervening sequences and no detectable concatemers. Our enzymatically produced oligonucleotides contained fewer truncated sequences compared with conventional synthetic oligonucleotides.

For this study, we amplified subpools from two different libraries for two proof-of-principle applications: DNA nanostructures and FISH.

### Folding DNA nanostructures

**Figure 4:**
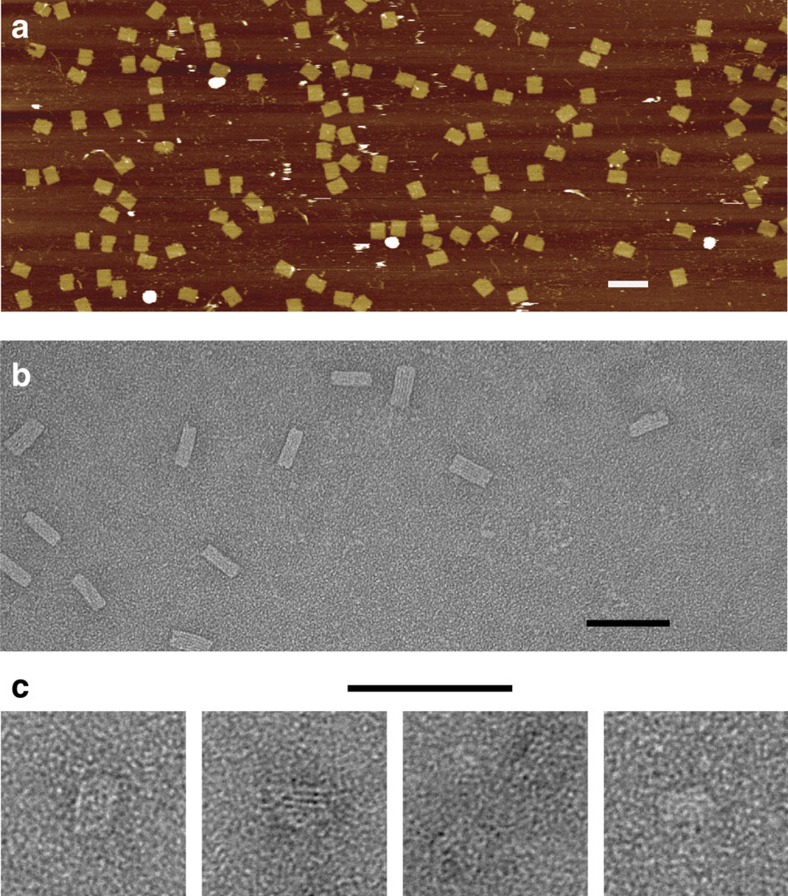
Structures folded from oligonucleotides amplified by circle-to-circle amplification. (**a**) An AFM image of a single-layer rectangular origami structure as in literature^1^. (**b**) A TEM image of a multilayer three-dimensional origami structure. (**c**) A small scaffold-free three-dimensional ‘DNA-brick'^5^ structure. Scale bars in **a**,**b**, 100 nm; in **c**, 50 nm. Panel **a** was folded with oligonucleotides from Fig. 3 lane 6; **b** with Fig. 3 lane 4.

The library from which we amplified the DNA structures contained ∼3,700 oligonucleotides in eight subpools, each as a one- or as a two-barcode version (design details in Supplementary Note 1). All subpools amplified strands with the expected length pattern (Supplementary Fig. 2 and comparison of enz and syn in Fig. 3). Oligonucleotides were purified using anion exchange chromatography (see Methods, Supplementary Note 3 and Supplementary Fig. 3) and folded at a 10-fold molar excess over scaffold strands (origamis Fig. 4a,b) or at 200 nM each (**c**). The folded structures are indistinguishable, using agarose-gel electrophoresis or atomic force microscopy (AFM) or transmission electron microscopy (TEM) imaging, from structures folded using synthetic oligonucleotides (Supplementary Fig. 4).

The DNA nanotechnology library contained oligonucleotides for the PCR amplification of the same target structures as for the c2ca designs. We used the same restriction enzymes (Nb.BsrDI and Nt.BspQI) that were encoded between the primers and the target sequences to cut the PCR primers out. Oligonucleotides recovered by denaturing PAGE from control PCR experiments did not succeed with the folding of DNA structures (data not shown). A reason for the failure might be the higher sequence-dependent amplification bias of PCR compared with c2ca^24^.

Moreover, we had to apply 10 times more nicking enzymes than in the c2ca experiments to produce the same amount of ss oligonucleotides, making this final step 10 times more expensive. Why partly single-stranded template (RCA product) was cut more efficiently by the nicking enzymes than double-stranded DNA (PCR product) is not clear and has, to the best of our knowledge, not been reported yet.

### FISH probes

FISH is a powerful single-cell assay that allows for the direct visualization of the *in situ* positioning of DNA and RNA molecules in a sequence-specific manner. Typically, FISH probes are produced from amplified inserts of genomic DNA, which are labelled via the random incorporation of fluorophore- or hapten-conjugated nucleotides and then fragmented by DNase I treatment into fragments of ∼100 base pairs^25^,^26^.

As these genomic inserts tend to range in size from tens of kilobases to hundreds of kilobases, the fragmentation step can produce a large and variable population of short DNA segments, many of which may not function optimally for FISH because of misincorporation of label, the presence of sequence complementary to repetitive elements or other off-target locations, or the presence of stable secondary structures.

The availably of high-complexity oligonucleotide libraries has led to the development of ‘next-generation' FISH probes derived from synthetic DNA; these probes have their sequences chosen bioinformatically, and thus can be designed to avoid repetitive elements and have desirable thermodynamic properties. The oligonucleotide libraries that encode these probes can either be directly labelled with fluorophore and used for FISH^14^ or instead serve as a renewable template for a PCR amplification step that produces products that are subsequently converted to randomly labelled^13^ or uniformly labelled ssDNA ‘Oligopaint' probes^15^.

**Figure 5:**
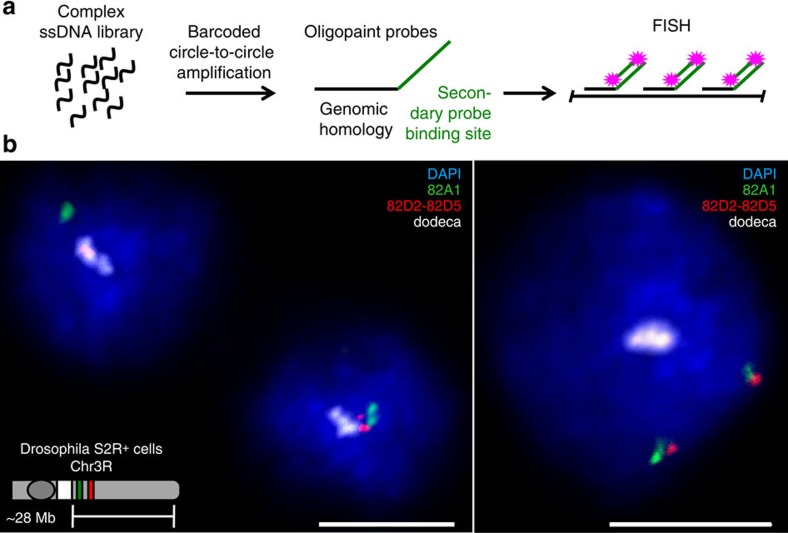
Highly efficient Oligopaint FISH with probe sets made by barcoded c2ca. (**a**) A cartoon that illustrates the structure of c2ca-amplified Oligopaint probes and the secondary oligonucleotide strategy^15^,^27^,^28^ used to recruit fluorescent label (**b**). Two confocal images of three-colour FISH performed in Drosophila S2R+ cells with a probe set of 679 oligonucleotides targeting 56 kb at 82A1 (green), a probe set of 719 oligonucleotides targeting 50 kb at 82D2-82D5 (red) and a single oligonucleotide targeting the highly repetitive dodeca percentromeric satellite sequence (white). The Oligopaint probes were visualized by the addition of fluorophore-labelled secondary oligonucleotides complementary to binding sequences encoded in the probe molecules (left panel: 82A1—ATTO565, 82D2-82D5—ATTO488; right panel: 82A1–6-FAM, 82D2-82D5—TYE563), while the dodeca probe carried a Cy5 direct label. Note that Drosophila pairs its homologous chromosomes in somatic cells; therefore, most cells are expected to have only one focus despite that fact that S2R+ cells are tetraploid for chromosome 3. Images are maximum Z projections. Scale bars, 5 μm.

Given the high efficiency at which c2ca can produce pools of ssDNA, we set out to explore the possibility of producing Oligopaint probes from libraries amplified by barcoded c2ca (amplification results in Supplementary Fig. 5). As the c2ca-amplified oligonucleotides do not carry a direct label, we applied a technology that has been developed to enhance Oligopaints that calls for a common binding site for a fluorophore-labelled ‘secondary' oligonucleotide (15,27,28refs 15,27,28; Fig. 5a). To test the efficiency of this strategy for c2ca, we designed a multiplexed library targeting a centromere-proximal portion of the right arm of Drosophila chromosome 3 (region 82A1-82D5). We then performed three-colour FISH in S2R+ cells with a probe set consisting of 679 oligonucleotides targeting a 56-kilobase region at 82A1, a probe set consisting of 719 oligonucleotides targeting a 50-kilobase region at 82D2-82D5, and a single Cy5-labelled oligonucleotide^29^ that targets the highly repetitive dodeca percentromeric satellite sequence. For all three probes, we observed crisp, clean signals with very low background (Fig. 5b). Furthermore, the staining was very efficient, with >94% of nuclei displaying at least one focus (94.3% for 82A1, 97.1% for 82D2-82D5 and 100% for dodeca; *n*=105; Table 1).

## Discussion

The gold standard in DNA amplification is PCR; however, the method is ill-suited for the scalable production of primer-free single-stranded oligonucleotides. We believe that our RCA-based method is superior for this purpose for several reasons. One reason is that the final concentration of the oligonucleotide copies in a RCA can be ∼15 times higher than in PCR^29^. The main reason for this is that, at high concentrations of double-stranded PCR products, they preferentially reanneal to themselves after denaturation and no further amplification is achieved. A second reason is that RCA is an isothermal process; PCR requires very quick temperature changes. This limits scalability to large reaction vessels so that many PCR reactions (for example, several 96-well plates) have to be combined instead. A third reason is that RCA directly produces ssDNA so that additional laborious and lossy steps such as preparative denaturing PAGE or expensive magnetic beads^21^ to separate the double strands can be omitted. A fourth reason is that the workup of single-stranded oligonucleotides produced by RCA is potentially automatable by denaturing HPLC. In contrast, separation of nicked double-stranded PCR products by denaturing HPLC was not successful and could only be achieved by labour-intensive denaturing PAGE. A fifth reason is that less ‘waste' material (that is, the reverse strand in PCR) that can be a cost factor in the long run is produced. A sixth reason is that the sequence-dependent amplification bias is lower for c2ca due in part to fewer rounds of amplification^24^. A seventh reason is that nicking reactions are ∼10 times more efficient and therefore cheaper with partly single-stranded templates than with PCR products.

In summary, no other currently available technology is suitable to produce tens of microgram of dozens or hundreds of subpools for a reasonable price. It could be of immediate use to others as only commercially available reagents, oligonucleotide libraries and standard equipment can be used for our method. A detailed comparison of our method with competing methods can be found in Supplementary Note 4.

We achieved a production of nanomole amounts (all oligonucleotides of a subpool combined) of purified oligonucleotides for a typical reagent cost of US$20 nmol^−1^ (Supplementary Notes 5 and 6 and Supplementary Fig. 6), which is ∼350 times cheaper than the recent method described in ref. 21. The cost is dominated by commercial enzymes (see Supplementary Table 1), and therefore a further cost reduction by 1 or 2 orders of magnitude seems feasible through in-house production of enzymes to meet a higher demand. The savings compared with synthetic oligonucleotides depend on the total number of oligonucleotides and the required amounts. The cost reduction is greatest when very many (hundreds or thousands) of oligonucleotides are needed at only picomole or nanomole amounts (per oligonucleotide). Synthetic oligonucleotides are still less expensive for larger-scale applications requiring, for example, nanomole amounts per oligonucleotide. The c2ca was carried out tens of times for the applications described herein and for several other oligonucleotides and oligonucleotide pools with consistent results.

Shorter or longer oligonucleotides than the ones from this study could also be produced with modified designs. For example, two to three target oligonucleotides could be encoded in one library strand similar to Ducani's protocol^23^, or variable spacers in the intervening sequences could be used to achieve templates of about the same length and to prevent any length-dependent amplification bias. In additional experiments, we confirmed that the amplification is robust enough for the parallel amplifications of many more sequences than in these studies (data not shown).

The method requires a total of a few hours of manual pipetting and handling steps spread out over 4 days (see Methods). The most time-consuming steps are programmable incubations (14 h) in a standard PCR cycler that need no attendence by the user. The incubation times were chosen to maximize yields and to match the diurnal schedule of experimentalists; the protocol could, however, also be carried out with much shorter incubation times (particularly the first two rounds and conveniently when automated) at only few per cent lower final yield (Supplementary Note 5) if time is more critical for certain applications.

Our method enables a variety of applications. For example, the design principles for successful DNA origami folding, for example, are not yet well understood. Currently, the synthetic oligonucleotides for each independent origami design costs around US$1,000. However, only a few picomoles of the typically several nanomoles received of each oligonucleotide are needed for a test folding. Our method, therefore, enables the prototyping of many different versions of a design for a fraction of the cost and a broad testing of design hypotheses comes into reach. For large-scale applications in nanotherapeutics or in material science, the best designs could then be scaled up with conventional synthetic oligonucleotides. Much larger structures than the current examples could also be prototyped at a fraction of the cost now, which enables the continuation of the current exponential complexity growth of the field. The FISH results demonstrate that Oligopaint probe sets produced by barcoded c2ca are robust and reliable tools for the study of the genome *in situ* enabling affordable genome-wide assays. Finally, applications in synthetic biology such as MAGE, gene and genome synthesis and any other research requiring thousands of high-quality oligonucleotides at subnanomole quantities (each) would receive a boost from a largely reduced oligonucleotide cost and an automatable production pipeline.

## Methods

### Materials

Enzymes and respective buffers were purchased from New England Biolabs (NEB) or Enzymatics (see Supplementary Table 1). Deoxyribonucleotide triphosphates were purchased from Enzymatics. Denaturing PAGE gels (15% TBE Urea gels, Invitrogen NuPage) were run at ∼55 °C. Gels were post-stained with SybrGold (Invitrogen). Chemicals were purchased from Sigma-Aldrich, and primers were purchased from IDT or Bioneer as desalted oligonucleotides and were used without further purification. The oligonucleotide library for the DNA nanoarchitecture experiments was kindly provided by Agilent produced on the sure-print platform. The library for the FISH experiments was purchased from LC Sciences.

### Detailed step-by-step protocol for three rounds of the c2ca

All buffers and enzymes are from NEB, unless otherwise indicated. All reactions and master mixes are prepared at room temperature (RT). Phosphorylation of the library is necessary for non-phosphorylated libraries before the first ligation. To do this, 25 μl of the crude oligonucleotide library are phosphorylated in a 50-μl reaction as follows: add 5 μl 10 × T4 DNA ligase buffer, 19 μl water and 1 μl T4 polynucleotide kinase (10 U μl^−1^); incubate at 37 °C for 30 min; and heat-inactivate at 65 °C for 20 min.

*First-round amplification*. To anneal the first-round primer, prepare a master mix to add the following per reaction to obtain a total volume of 9 μl: 0.2 μl of the phosphorylation reaction from above, 0.98 μl 10 × T4 DNA ligase buffer and 7.82 μl water. Add 1 μl 300 nM first-round primer to achieve a total volume of 10 μl. Incubate 2 min at 65 °C and cool to RT at −2 °C min^−1^. For ligation, prepare a master mix to add 5 μl to each reaction consisting of 4.4 μl water, 0.5 μl 10 × T4 DNA ligation buffer, 0.1 μl T4 DNA ligase (120 U μl^−1^) and thereby achieve a final volume of 15 μl per reaction. Incubate 20 min at RT and heat-inactivate 10 min at 65 °C. For RCA, prepare a master mix to add 5 μl to each reaction consisting of 3.7 μl water, 0.5 μl 10 × phi29 buffer, 0.08 μl 1 M dithiothreitol (DTT), 0.2 μl 100 × BSA, 0.3 μl dNTP mix (25 mM each)*, 0.2 μl phi29 DNA polymerase (10 U μl^−1^) and thereby achieve a total volume of 20 μl per reaction. Incubate 14 h at 30 °C and heat-inactivate for 10 min at 65 °C, 4 °C until next reaction.

*Second-round amplification*. To anneal the second-round primer, add 2 μl of 10 μM appropriate oligonucleotide to achieve a total volume of 22 μl per reaction. Incubate for 2 min at 95 °C, −0.1 °C s^−1^ to 70 °C, 70–60 °C at −1 °C min^−1^ and to RT at −0.1 °C s^−1^. To digest with HindIII, prepare a master mix to add 7 μl to each reaction consisting of 5.97 μl water, 0.7 μl NEBuffer 2, 0.029 μl 1 M DTT, 0.3 μl HindIII (20 U μl^−1^) and thereby achieve a total volume of 29 μl per reaction. Incubate 1 h at 37 °C; heat-inactivate for 20 min at 65 °C; cool to 20 °C at −0.1 °C s^−1^. For ligation, prepare a master mix to add 7 μl to each reaction consisting of 2.74 μl water, 0.7 μl 10 × ligation buffer, 2.9 μl 10 mM ATP, 0.36 μl 1 M DTT, 0.3 μl T4 DNA ligase (120 U μl^−1^) and thereby achieve a total volume per reaction of 36 μl. Incubate 20 min at RT, heat-inactivate for 10 min at 65 °C and cool to 20 °C at −0.1 °C s^−1^. For RCA, prepare a master mix to add 15 μl to each per reaction consisting of 11.17 μl water, 1.5 μl 10 × phi29 buffer, 0.204 μl 1 M DTT, 0.51 μl 100 × BSA, 0.86 μl dNTP mix (25 mM each)* and 0.8 μl phi29 DNA polymerase. Incubate 14 h at 30 °C and heat-inactivate for 10 min at 65 °C, 4 °C until next reaction.

*Third-round amplification*. To anneal the third-round primer, add 6 μl of 100 μM the appropriate oligonucleotide to achieve a total volume of 57 μl per reaction. Incubate 2 min at 95 °C, −0.1 °C s^−1^ to 70 °C, 70–60 °C at −1 °C min^−1^ and to RT at −0.1 °C s^−1^. For HindIII digestion, prepare a master mix to add 27 μl to each reaction consisting of 22.3 μl water, 2.7 μl NEBuffer 2, 0.84 μl 1 M DTT, 2 μl HindIII (20 U μl^−1^) and thereby achieve a total volume of 84 μl per reaction. Incubate 1 h at 37 °C; heat-inactivate for 20 min at 65 °C; and cool to 20 °C at −0.1 °C s^−1^. For ligation, prepare a master mix to add 16 μl to each reaction consisting of 3.8 μl water, 1.6 μl 10 × ligation buffer, 8.4 μl 10 mM ATP, 1 μl 1 M DTT, 1.2 μl T4 DNA ligase (120 U μl^−1^) and thereby achieve a total volume per reaction of 100 μl. Incubate 20 min at RT; heat-inactivate 10 min at 65 °C; and cool to 20 °C at −0.1 °C s^−1^. For the final RCA, where a large amount of material will be produced, transfer 50 μl of each reaction to a deep-well plate or 1.5 ml reaction vial. Prepare master mix to add 200 μl to each reaction consisting of 160.75 μl water, 20 μl 10 × phi29 buffer, 1.25 μl 1 M DTT, 2.5 μl 100 × BSA, 8 μl dNTP mix (25 mM each)* and 7.5 μl phi29 polymerase. Incubate for 14 h at 30 °C and heat-inactivate 10 min at 65 °C, 4 °C until next reaction. To anneal nicking primers, add 50 μl of 100 μM each of two appropriate oligonucleotides to achieve a total volume of 350 μl per reaction. Incubate 2 min at 95 °C, −0.1 °C s^−1^ to 70 °C, 70–60 °C at −1 °C min^−1^ and to RT at −0.1 °C s^−1^. For the double-nicking reaction, prepare a master mix to add 325 μl to each reaction consisting of 244.4 (194.4) μl water, 37.5 μl 10 × NEBuffer 2, 3.15 μl 1 M DTT, 20 μl Nt.BspQI, 20 μl Nb.BtsI and thereby achieve a total volume per reaction of 675 μl. Incubate 12 h at 50 °C and heat-inactivate 5 min at 95 °C, 4 °C till workup. The reaction after this step is a cloudy suspension due to denatured proteins.

Centrifuge for 20 min at 14,000*g* to pellet the denatured proteins. Oligonucleotides and short DNA fragments stay dissolved in the supernatant. Alternatively, the suspension can be filtered through a 0.2-μm filter to remove denatured proteins.

The entire amplification takes 4 days but is governed by long reaction times. Hands-on time is limited to the addition of primers (can be performed with multichannel pipettes from 96-well plates) and the preparation and the addition of the master mixes. All steps can potentially be automated for larger-scale applications.

* Most recent experiments suggest that a total concentration of 0.4 mM dNTPs (each) and 3 h amplification times are sufficient for RCA (Supplementary Note 7 and Supplementary Fig. 7).

### Anion exchange HPLC

Dionex (Thermo Fisher) DNA swift columns (150 × 50 mm) were used on an Agilent 1200 series HPLC system at 65 °C and a flow rate of 1 ml min^−1^. We let Solvent A be 20 mM Tris-HCl (pH 8 at 20 °C) and let Solvent B be Solvent A+1.25 M guanidinium hydrochloride. Our standard gradient, in terms of per cent solvent B, was as follows: 0–1 min: 5%; 4 min: 40%; 16 min: 60%; 18–20 min: 100%; 21–24 min: 5%. Fractions were automatically collected in 96-deep-well plates by a fraction collector. Fractions containing the desired oligonucleotide lengths were pooled and worked up together. Typically, fractions between 8 and 12 min contained oligonucleotides between 25 and 50 nt. The collected solution can be either ethanol precipitated or worked up with silica columns. Ethanol precipitation typically gave ∼20% higher final yields; however, the workup with silica coulumns is still attractive as it can be automated more easily.

### Barcode design

Barcodes were designed to have no secondary structures with the rest of the intervening region or secondary structures with a melting temperature of less than 37 °C at 10 mM MgCl_2_ calculated with the IDT OligoAnalyzer (idtdna.com; models based on unafold).

### DNA nanostructure experiments

After the last double-nicking step, oligonucleotides were purified using anion exchange chromatography and ethanol precipitation. The strands were redissolved in 5 mM TE buffer (pH 8.0) and the concentration of the library was determined photometrically. A 10-fold molar excess of staple strands over scaffold strands was used for DNA origami structures. p8064 was used for the 48-helix bundle; a commercial M13 plasmid (NEB) was used for the planar rectangle origami. Single-stranded DNA-brick structures were folded at 200 nM per strand in TE-Mg buffer (5 mM Tris, 1 mM EDTA pH 8.0 and 40 mM MgCl_2_). The planar structure was folded with 12.5 mM MgCl_2_ and the 48-helix bundle with 16 mM MgCl_2_. The planar rectangle was folded from 80 to 20 °C at −1 °C min^−1^. The other two structures were folded from 80 to 65 °C at −1 °C per 5 min and from 65 to 20 °C at −1 °C per 20 min.

The folding products were separated by agarose gel electrophoresis and stained by SybrSafe (Invitrogen). The bands containing the folded product were cut out, eluted by centrifuging the gel piece for 5 min at 1,000*g* through a freeze ‘n squeeze column (Bio-Rad) and imaged on a JEOL JEM-1400 TEM. The scaffold-free single-stranded brick structure was prepared in the same way. The planar rectangular origami structure was imaged without further purification on mica in liquid tapping mode on a Veeco Multimode 8 with Veeco DNPS tips.

### FISH experiments

FISH was performed as described previously^15^,^27^. Briefly, 10–40 pmol of primary c2ca-amplified Oligopaint probes and 40–50 pmol of secondary oligonucleotide were added simultaneously to a 25-μl hybridization in the presence of RNase. Samples were denatured for 2.5 min at 92 °C and then allowed to hybridize overnight at 42 °C. Slides were then washed for 15 min at 60 °C in 2 × SSCT, and then for 10 min at RT in 2 × SSCT, then for 10 min at RT in 0.2 × SSC and finally mounted in Slowfade Gold mounting media with 4,6-diamidino-2-phenylindole (Invitrogen). Samples were imaged on Olympus IX-83 using a × 60, NA 1.42 lens and an Olympus XM-10 camera or a Zeiss LSM780 laser scanning confocal microscope using a × 63, NA 1.40 lens. Images processing was performed with the Olympus CellSens Dimension or Zeiss ZEN microscope-specific software as well as Adobe Photoshop. For the quantification of the number of FISH signals per nucleus, signals <1 μm apart were considered to be a single focus.

## Additional information

**How to cite this article:** Schmidt, T. L. *et al.* Scalable amplification of strand subsets from chip-synthesized oligonucleotide libraries. *Nat. Commun.* 6:8634 doi: 10.1038/ncomms9634 (2015).

## Supplementary Material

Supplementary Information Supplementary Figures 1-7, Supplementary Table 1 and Supplementary Notes 1-7

## Figures and Tables

**Table 1:** FISH data.

**Probe Set**	**Span kb**	**Complexity**	**Chr.**	**Start**	**Stop**	***n***	**0 Foci**	**1 Focus**	**2 Foci**	**>2 Foci**	**% Labelling**
82A1	56	679	3R	60,018	116,183	105	3	70	29	3	97.1
82D2-82D5	50	719	3R	559,697	609,617	105	6	79	19	1	94.3
dodeca	Unknown	1	3R	N/A	N/A	105	0	51	43	11	100

Chr., chromosome; FISH, fluorescence *in situ* hybridization; N/A, not applicable.

For each probe set, the genomic span in kilobases, the number of oligonucleotides in the probe set (complexity), the Chr. and start and stop of the span, the number of nuclei imaged (*n*), the number of nuclei in which 0, 1, 2 or >2 signals were observed and the % of nuclei with >1 focus (% labelling) are given.
