# Supplementary material for: Scalable amplification of strand subsets from chip-synthesized oligonucleotide libraries
Source: Nat Commun. 2015 Nov 16;6:8634. doi: 10.1038/ncomms9634 (PMC4660042; doi:10.1038/ncomms9634)
Supplement: Supplementary Information — Supplementary Figures 1-7, Supplementary Table 1 and Supplementary Notes 1-7 [file ncomms9634-s1.pdf]

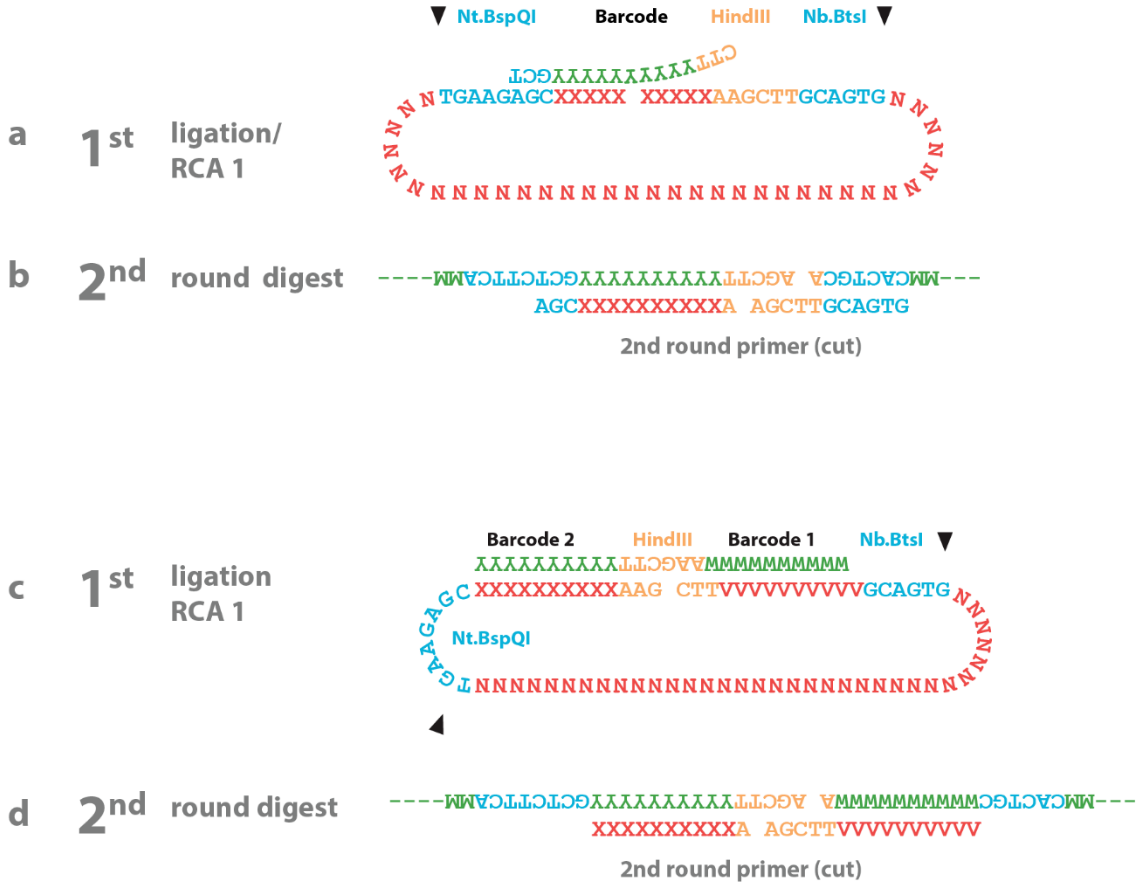

Supplementary Figure 1: One vs. two barcode designs. (**a-b**) one-barcode design; (**c-d**) two-barcode design. (**a**) and (**c**) show the hybridization of the first round primer before the first ligation step. (**b**) and (**d**) show the fragments created after the second restriction digest. “X” and “Y”, “V” and “W”, and “N” and “M” are complementary.

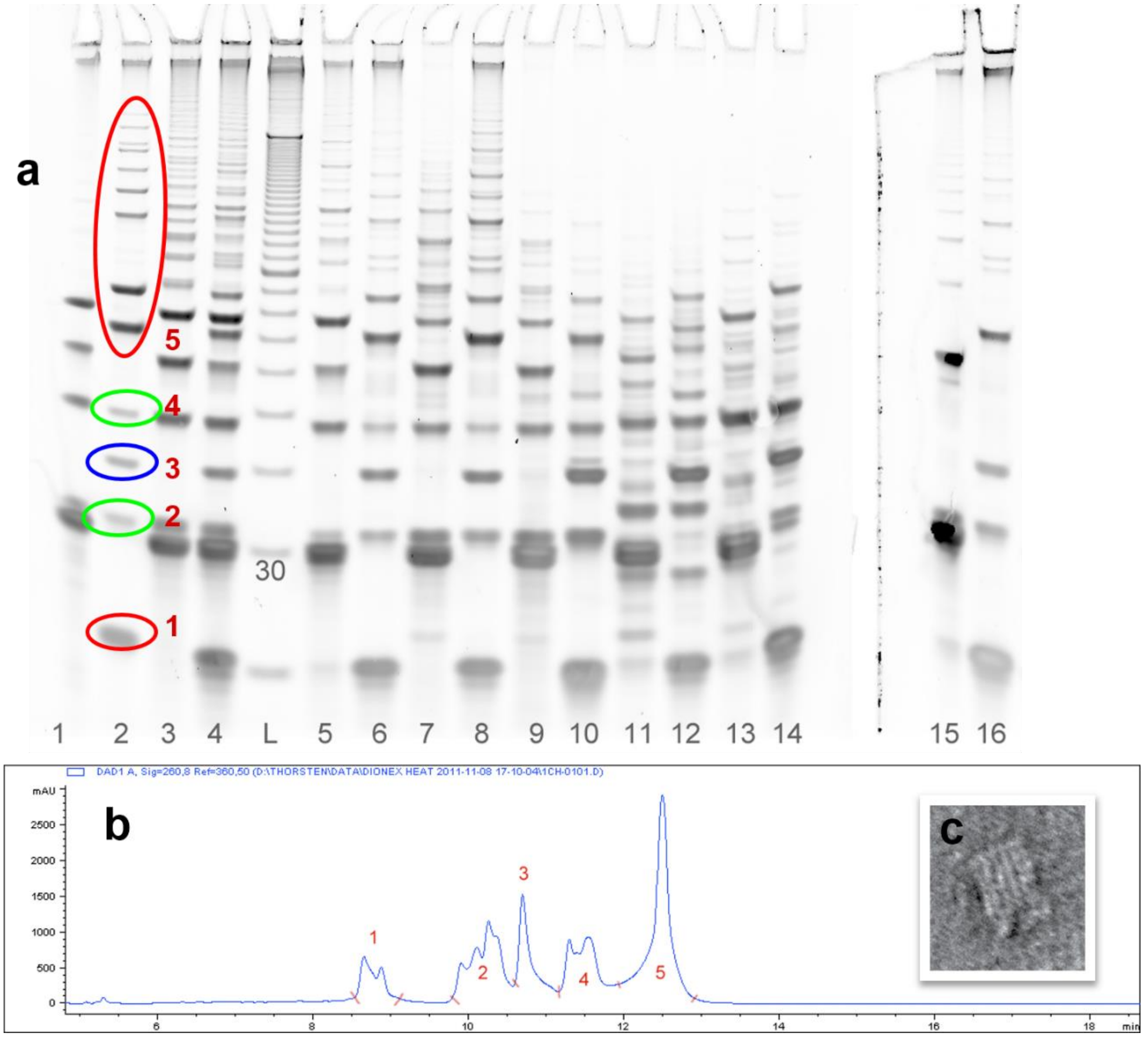

Supplementary Figure 2: Workup of a double nicking reaction. (**a**) Analytical PAGE of 16 crude double nicking reactions. In these reactions, the double nicking reaction did not go to completion. Typically bands longer than 50 nt correspond to incompletely cut concatemers. Lanes with odd numbers are one-barcode designs, lanes with even numbers two-barcode designs. The gel demonstrates, that all 16 amplification reactions produced a comparable amount of oligonucleotides. The reactions 2 and 4 contained oligonucleotides for a scaffold-free structure (**c**); 13 and 14 contained the 48-helix bundle structure, and 15 and 16 contained the planar rectangular origami structure. (**b**) HPLC trace at 260 nm of subpool nicking reaction 2. Fraction 1 contains the excess of the two split nicking primers; fraction 2 contains the 32 nt target oligonucleotides, fraction 3 the excised intervening sequence, fraction 4 contains the 48 nt target oligonucleotides; and fraction 5 all uncut concatemers. (**c**) Is a TEM image from a scaffold-free single-stranded tile structure folded with fractions 2 and 4 and synthetic oligonucleotides. Even though all oligonucleotides in a fraction have the same length, they can have slightly different retention times due to a different base composition giving rise to multiple peaks observed in the chromatogram.

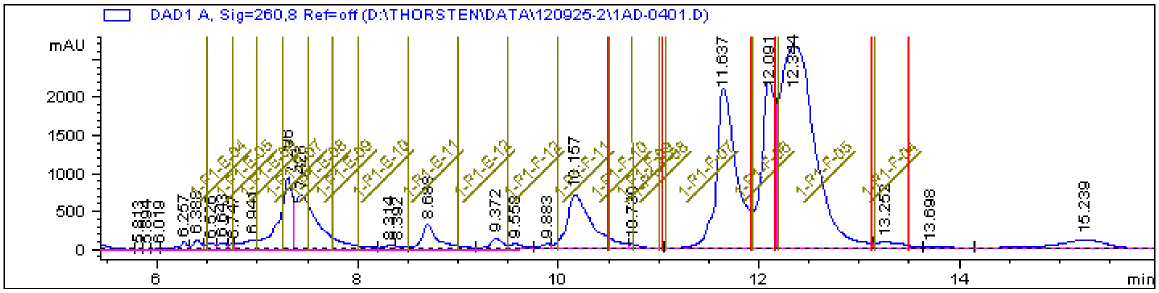

Supplementary Figure 3: HPLC trace of the workup of the staple strands for the 48-helix bundle structure in **Fig. 4b**. The green lines indicate the fractions collected automatically by the fraction collector. Here, most of the intervening sequence is present as a complex with the excess of split nicking primers in the peak at 11.6 minutes, the excess of nicking primers were in the fractions between 7 and 8 minutes. Fractions E10-F11 were pooled and ethanol precipitated. An aliquot was loaded to lane 4 in **Fig. 3**. With another aliquot, a folding reaction was performed (**Supplementary Note 4** and **Fig. 4b**). In this experiment, 1,0 nmol of staple strands were recovered (which was the lowest amount in this set of experiments), other purifications yielded up to 4 nmol.

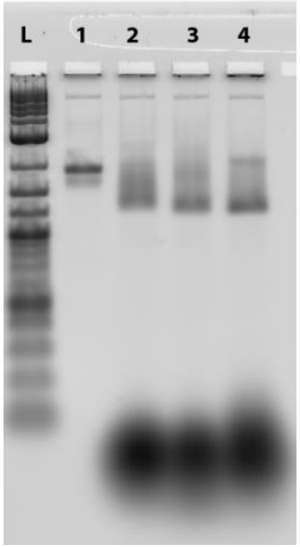

Supplementary Figure 4: Native agarose gel (1.5 %) of the 48-helix bundle folding reaction. (**L**) 2 log ladder (NEB); (**1**) scaffold (p8064); (**2**) folded one-barcode c2ca product (anion-exchange HPLC purified); (**3**) folded 2-barcode c2ca product (also anion-exchange HPLC purified); (**4**) control folding reaction with synthetic oligonucleotides.

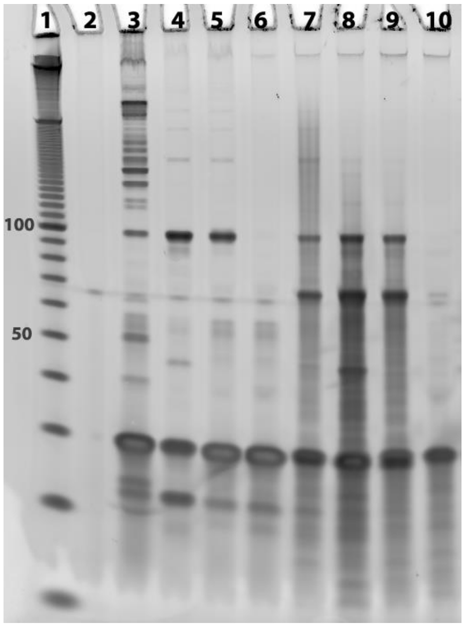

Supplementary Figure 5: Analytical PAGE gel of the amplification for the FISH experiments. Lane **1**: 10 bp marker; lane **2**: an aliquot of the crude oligonucleotide library contained too little material to show up in the pel (else, a band at 100 nt should be visible). (**3-6**) 2 µl aliquots of the

last HindIII digest, (**7-10**) analysis of the crude double nicking reaction before workup. Bands at 70 nt are the primary FISH probes, bands at 100 nt are FISH probes plus 30 nt intervening sequence. (**8**) was probe set 82D2-82D5; (**9**) contained probe set 82A1. For the FISH experiments, the crude double nicking reaction was purified by HPLC and the 70 nt products were collected. Subpool (**6**/**10**) did not amplify (presumably due to a pipetting error).

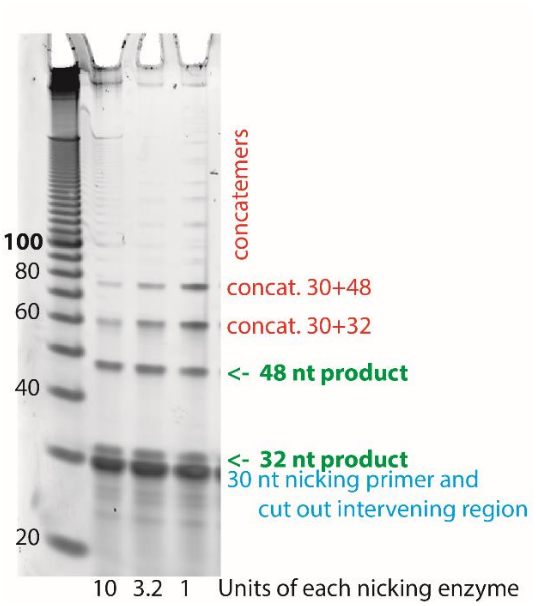

Supplementary Figure 6: Analysis of the double nicking digest with varying amounts of nicking enzymes. The two product band (32 nt and 48 nt) are the desired products, some concatemers remain in all 3 digests.

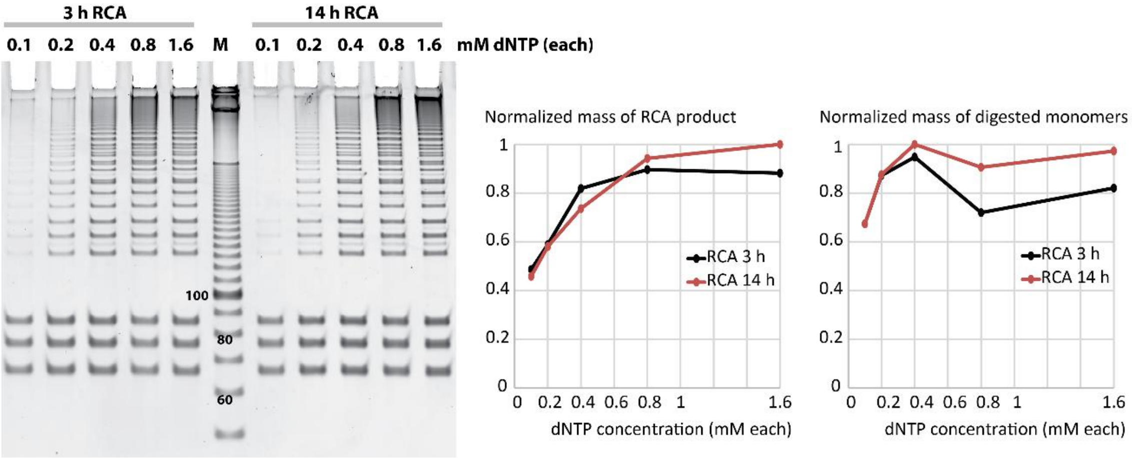

Supplementary Figure 7: Influence of the dNTP concentration on the RCA yield. (Discussion below)

reagent	supplier	[µl]	USD	µl used	US$ used	% of cost
Kinase (10 U/µl)	NEB	50	54	0.05	0.1	0.2
T4 Ligase (120 U/ µl)	Enzymatics	800	250	2	0.5	1.5
ATP (10 mM)	Enzymatics	5,000	100	11.8	0.2	0.7
HindIII (20 U/µl)	NEB	500	216	2	1.0	3.0
Phi 29 Polymerase (10 U/µl)	Enzymatics	200	250	9	11.1	31.8
dNTPs (25mM each)	Enzymatics	4,000	250	10	0.6	1.7
Primers	bioneer/ IDT		0		0.0	0,0
Nt.BspQI (10 U/µl)	NEB	500	256	21	10.6	30.6
Nb.BsrDI (10 U/µl)	NEB	500	256	21	10.6	30.6
				**sum**	**34,8**	**per rcn**

Supplementary Table 1: Cost calculation

Supplementary Note 1: One or two-barcode design?

In this work, we tested two different template strand designs: One with one and one with two 10 nt barcodes per intervening sequence (**Supplementary Fig. 1**). The rationale for one barcode was to keep the length of the template oligonucleotides as short as possible, because the accumulated sequence errors increase with increasing length during oligonucleotide synthesis. Two barcodes on the other hand increase the sequence space for different barcodes.

The two-barcode design has several advantages over the one-barcode design:

1) Structures shown in **Figure 4** were folded from oligonucleotides amplified with the two-barcode design. Both designs amplified oligonucleotides that could be folded, however the oligonucleotides amplified from the two-barcode designs folded more reliably and with slightly fewer defects (**Supplementary Fig. 4** lane 3 and in AFM or TEM images, not shown). A potential reason for this could be that primers in the one-barcode design are shorter. For the first round primer they are 16 nt vs 26 nt. The second round primers were about the same length (25 and 26 nt), but for the religation of the first concatemer (**Supplementary Fig. 1b** and **d**), the overlap with the upstream (**b**, top right) sequence is very short (only 7 nt). Some staple strands within the subpool might form secondary structures and the weak 7 nt interaction might not be enough to overcome this. As a result, not all sequences would be ligated and amplified.

2) The intervening sequence is longer and can be removed more easily by HPLC chromatography (discussed in **Supplementary Note 3**). This is particularly true where short oligonucleotides (such as 25-mers) were produced. For these, a larger retention time difference of short incompletely nicked oligonucleotides (e.g. 25 nt + the intervening sequence) from the longest target sequence (e.g. 52 nt + intervening sequence) is desirable.

3) For the 2-barcode design, we also tested nicking primers consisting of 2 halves, the Nb.BsrDI nicking primer and the Nt.BspQI nicking primer. The split nicking primers were only 20 nt long and the excess of primers could be more easily separated by anion exchange chromatography from target sequences (typically between 26-48 nt) than the 40 nt full-lengh nicking primer.

4) The intervening sequence is cut close to the center (**Supplementary Fig. 1d**) after the second round restriction digest, whereas the one-barcode version is cut close to the transition to the target sequence (MMM sequence in **b**). This may lead to a less efficient religation for the third and final RCA step because of the low melting temperature of the short fragment.

For the FISH experiments, however, we chose the one-barcode design to keep the length of the template at 100 nt (recommendation from LC Sciences).

Moreover, we tested different nicking enzymes cutting the upstream strand (Nb.BtsI and Nb.BsrDI), but found no significant differences in the performance of the two enzymes in the digest of RCA products.

Supplementary Note 2: Oligonucleotide and primer sequences

Oligonucleotide libraries:

To generate the sequences for a library order, we attached barcode and restriction enzyme sequences to the reverse complement of the target sequence as in **Supplementary Fig. 1**.

Two different oligonucleotide libraries were used for the structural DNA nanotechnology experiments and the FISH experiments, respectively. The first library contained 3,696 different sequences, the second contained 3,918 sequences. The sequence files can be obtained from the authors. The nanotechnology library also contained template strands for the amplification by PCR. Many sequences of the libraries were not included in this study and can therefore be considered background.

Primer sequences:

Structure	Primer	Sequence

48 hb (1 bc)	first round primer	**CTTATCCCGCAGAGCT**
48 hb (1 bc)	second round primer	**TGAAGAGCTCTGCGGGATAAGCTTGCAATG**
48 hb (1 bc)	third round primer	**CATTGCAAGCTTATCCCGCAGAGCTCTTCA**
48 hb (2 bc)	first round primer	**ATACTGGAGTAAGCTTTAACTCTCGG**
48 hb (2 bc)	second round primer	**CCGAGAGTTAAAGCTTACTCCAGTAT**
48 hb (2 bc)	Nb.BsrDI nicking primer	**GCTTACTCCAGTATGCAATG**
48 hb (2 bc)	Nt.BspQI nicking primer	**TGAAGAGCCCGAGAGTTAAA**
rectangle (1 bc)	first round primer	**CTTAGCGATTCAGGCT**
rectangle (1 bc)	second round primer	**TGAAGAGCCTGAATCGCTAAGCTTGCAATG**
rectangle (1 bc)	third round primer	**CATTGCAAGCTTAGCGATTCAGGCTCTTCA**
rectangle (2 bc)	first round primer	**GTGACCTTTGAAGCTTAACAAACGGA**
rectangle (2 bc)	second round primer	**TCCGTTTGTTAAGCTTCAAAGGTCAC**
rectangle (2 bc)	Nb.BsrDI nicking primer	**GCTTCAAAGGTCACGCAATG**
rectangle (2 bc)	Nt.BspQI nicking primer	**TGAAGAGCTCCGTTTGTTAA**
6x6x64 X (2bc)	first round primer	**CTAGCCTCACAAGCTTTAACCGTCAT**
6x6x64 X (2bc)	second round primer	**ATGACGGTTAAAGCTTGTGAGGCTAG**
6x6x64 X (2bc)	Nb.BsrDI nicking primer	**GCTTGTGAGGCTAGGCAATG**
6x6x64 X (2bc)	Nt.BspQI nicking primer	**TGAAGAGCATGACGGTTAAA**
6x6x64 Y (1 bc)	first round primer	**CTTCTGTCTGACAGCT**
6x6x64 Y (1 bc)	second round primer	**TGAAGAGCTGTCAGACAGAAGCTTGCAATG**
6x6x64 Y (1 bc)	third round primer	**CATTGCAAGCTTCTGTCTGACAGCTCTTCA**
6x6x64 Y (2 bc)	first round primer	**GCTGAGATGAAAGCTTCATACGGAGA**
6x6x64 Y (2 bc)	second round primer	**TCTCCGTATGAAGCTTTCATCTCAGC**
6x6x64 Y (2 bc)	Nb.BsrDI nicking primer	**GCTTTCATCTCAGCGCAATG**
6x6x64 Y (2 bc)	Nt.BspQI nicking primer	**TGAAGAGCTCTCCGTATGAA**

Note that second round primers of the one-barcode design are identical to nicking primers after the final round of rolling circle amplification. “48 hb” is a so-called multi-layered 48-helix bundle origami structure (**Fig. 4 b**); 6x6x64 is a scaffold-free DNA brick structure (adapted from Ref. [5]).

Primers for the FISH library:

The library for the FISH probes was ordered from LC Sciences in the one-barcode design to keep the final length of the sequences at 100 nt (70 nt target sequences + 30 nt intervening sequences). Note that the second round primers are identical to the nicking primers.

Pool 1 first round primer	CTTTTTTATAGTGGCT
Pool 2 first round primer	CTTCCCCATTATTGCT
Pool 1 second round primer	TGAAGAGCCACTATAAAAAAGCTTGCAGTG
Pool 2 second round primer	TGAAGAGCAATAATGGGGAAGCTTGCAGTG
Pool 1 third round primer	CACTGCAAGCTTTTTTATAGTGGCTCTTCA
Pool 2 third round primer	CACTGCAAGCTTCCCCATTATTGCTCTTCA

Supplementary Note 3: HPLC workup

Supplementary Figure 2a shows a gel of 16 amplification reactions, where concatemers are seen in all reactions. Removing these concatemers was the main purpose of HPLC workup. We did not find conditions, where the double nicking reaction was complete such that no residual concatemers remained (see **Supplementary Note 6**).

For the HPLC workup, guanidinium was chosen as a cation because it is a slightly denaturing (chaotropic) cation. Sodium is the most common cation for anion exchange chromatography but stabilizes DNA duplexes. To achieve fully denaturing conditions with a NaCl buffer, the column had to be heated to 85 °, which decreases its lifetime considerably and more DNA damage can be expected than at lower temperatures. With guanidinium chloride denaturing conditions were obtained at lower temperatures and with a comparable resolution.

The HPLC trace in **Supplementary Fig. 2 b** shows a fully denaturing experiment, where primers and intervening sequence elute as single-stranded DNA without forming a duplex. To achieve fully denaturing conditions, the crude material can be desalted and the temperature could be increased (e.g. to 85 °C).

Partially stabilizing the duplex by lower temperatures or by precipitating the crude reaction in the presence of 10 mM $MgCl_2$ allows stable double strands such as the complex between intervening sequence and nicking primer(s) to stay double-stranded and have retention times comparable to 60 nt (single barcode) or 80 nt (two barcodes). This way, most of the unwanted intervening sequence can be removed more easily from staple strands (**Supplementary Fig. 3** and **Fig. 3** lane **4**)

Supplementary Note 4: Comparison with other methods

Our method compares favorably with other published amplification methods. In this note we summarize the advantages of the method presented herein over competing methods.

Advantage over **strand displacement amplification** (Marchi, A. N., Saaem, I., Tian, J. & LaBean, T. H. One-Pot Assembly of a Hetero-dimeric DNA Origami from Chip-Derived Staples and Double-Stranded Scaffold. ACS Nano 130109093314009 (2013)). doi:10.1021/nn302322j):

- billionfold instead of only 4-fold amplification rates

Advantages over **PCR/ nicking/ PAGE elution**:

- single-stranded oligonucleotides are produced directly
- no fast temperature cycling is necessary as the amplification is isothermal
- final product concentration and therefore yield is at least 10-50 times higher
- Therefore larger reaction volumes such as 2 ml deep well plates are possible, each well producing more DNA as a combined 96-well PCR reaction.

Advantages over **strand displacement + PCR** (Marchi AN, Saaem I, Vogen BN, Brown S, LaBean TH. Toward larger DNA origami. Nano Lett. 14, 5740–5747, 2014):

- all advantages over PCR mentioned above
- no expensive streptavidin beads are required to generate ss DNA from PCR product
- works with commercially available libraries whereas LaBean's method requires custom-made chips produced by specialized lithography
- we produced 10–40 times more material per reaction (tens of μg instead of 1 μg)
- In LaBean's paper it was mentioned that their method is 1 order of magnitude cheaper than ordering commercial oligonucleotides (US$ 7000/10 => $700/μg). With our method we are over 2 orders of magnitudes cheaper than that (~ten μg for $20= $2/μg after purification).

Advantages over **monoclonal rolling-circle** method (Ducani, C., Kaul, C., Moche, M., Shih, W. M. & Högberg, B. Enzymatic production of 'monoclonal stoichiometric' single-stranded DNA oligonucleotides. Nature Methods 10, 647–652, 2013):

- no gene synthesis
- no cloning
- no sequencing
- thousands of sequences instead of only tens of sequences can be produced
- several orders of magnitude cheaper and faster

Supplementary Note 5: Yields and costs

Starting from attomoles of circular templates, we determined that an 8-hour RCA reaction can yield up to ~9400 copies of templates of 72 nucleotides, determined by quantitative real-time PCR. The last rolling circle amplification (and in some experiments even the second round) however yields a viscous, gelatinous solution, which is very difficult to pipette as concatemers tangle up. This also makes it impossible to aliquot a RCA reaction that reaches these high concentrations. Experimenting with different reaction conditions revealed an upper limit of around 20 μM copies of typical 30–50-mers in a RCA reaction.

This is in good agreement with the findings of Dahl.[24] The third round is therefore not limited by the amount of template or dNTPs, but mainly by the upper concentration limit of concatemer copies. The mechanism for this is not clear but may be due to sterical hinderance of tangled concatemers. Due to the gelatinous nature of the RCA products, the concentration of concatemers could only be determined by one of two methods:

1) Through the digestion of a final nicking reaction and the workup with a silica column (e.g. Zymo Research Oligo clean & concentrator), the OD 260 measurement of the recovered workup and subtraction of the nicking primer absorption or

2) Shearing of a RCA product by a focused Ultrasonificator (Covaris). Settings recommended for 800 bp fragments were applied and the sheared products were worked up with a PCR purification kit (Fermentas) and the concentration of the fragments was determined photometrically.

Both methods confirmed the upper amplification limit of 15–20 μM for a 72-mer circular template.

When scaling up the volumes of the last RCA and nicking reaction by factors of 2–4 from the same last religation reaction, ~2–4 times more final product was produced. As rolling circle amplification is, unlike PCR, an isothermal reaction, the final reactions could most likely be scaled up to be performed in mL quantities to yield high nmol or even mg quantities of oligonucleotides. For this reason we used only a fraction of the last religation reaction material for the final RCA, so that only the last RCA reaction could be repeated if more material was required.

Supplementary Table 1 shows the amplification cost for a reaction in a scale described in the Methods section, typically yielding 1–3 nmol of oligonucleotides after workup. The green/ red color code indicates relative costs per component, red being the most expensive. The table reveals that the most expensive reagents are Phi 29 Polymerase (7.5 µl are used in the last round amplification) and the two nicking enzymes used for the last double nicking reaction. In-house production of these components may reduce the amplification cost about one order of magnitude. Primer costs are omitted here as only a small aliquot of a commercial oligonucleotide synthesis is needed for one amplification. The same set of primers can

be used for amplifications from different libraries. The cost of the libraries are also omitted. The current cost is about 0.1 cents/ base in commercial oligonucleotide libraries. We expect this price to drop substantially as the production cost of oligonucleotide libraries is not dominated by chemical costs as in standard column-based oligonucleotide synthesis.

Supplementary Note 6: Attempts to achieve complete nicking digest

Initially we sought to achieve a complete double nicking digest after the final RCA reaction. This would have made the HPLC workup superfluous and would maximize the yield. However we found that the double nicking reaction could not be driven to completion with a reasonable amount of nicking enzymes. Supplementary Figure 6 shows an analytical PAGE gel of a double nicking digest with varying amounts of nicking enzymes. In this experiment, an oligonucleotide pool purchased from Invitrogen was circularized and subjected to one round of rolling circle amplification to mimic the final RCA round. This pool is identical to the one in lane 3 in **Supplementary Figure 2a** and the target oligonucleotides can be used fold a scaffold-free single-stranded tile structure in **Supplementary Figure 2c**. The library contained the 1-barcode design, where the intervening sequence consisted of 30 bp as shown in **Supplementary Figure 1a**. The Nicking primer was a full complement (30 nt) of the intervening sequence. For each double nicking reaction, 3.6 µl of a RCA reaction were annealed to 50 pmol of nicking primer and subjected to 2 h of digests in 5 µl reactions with 10; 3.2 or 1 U of each nicking enzyme. The gel results indicate that even at the highest concentration of nicking enzymes, which was 4.5 times higher than the one we typically used for the digest of the last round c2ca reactions (2,8 U/µl of test RCA; in last RCA digest 0,8 U/µl), some concatemers remained. The amount of nicking enzymes we selected for the final double digest seemed a good tradeoff between high digestion yield and cost.

This data suggests that a 100% cannot be reached. We do not have a satisfactory explanation for this behavior and also increasing the reaction times to 16 hours did not did not eliminate concatemers. Unfortunately we found that residual concatemers present in the oligonucleotide pool would impede the folding of both scaffold-free single-stranded tile structures and DNA origami. Moreover, excessive nicking primers, which were always added in a large excess, may be problematic in certain applications. We therefore decided to limit the usage of nicking enzymes to 0.8 U/µl of final RCA reaction and increase the reaction times to 16 h where the desired product are already the main product and to remove the remaining concatemers and nicking primers by anion exchange HPLC (**Supplementary Note 3**). In applications such as FISH, where residual concatemers or nicking primers may be tolerated, one may skip the HPLC step.

Supplementary Note 7: The influence of the dNTP concentration on the RCA yield

Initially, we did not test different concentrations of dNTPs as we had no indication that the amplification yield in our protocol is suboptimal. Our yield exceeds the 15 µM copies reported by Dahl et al in 2004 (see **Supplementary Note 5**), especially when accounting for losses of material due to incomplete digest (**Supplementary Note 6**) and during the final workup steps. Our rationale for increasing the dNTP concentration was to avoid a potential amplification bias in subpools where the 4 bases are unequally represented and where a substantial amount of the dNTPs is used up due to a high amplification rate.

On the other hand, an elevated dNTP concentration could inhibit the polymerase efficiency and therefore we tested the RCA efficiency as a function of the dNTP concentration between 0.1 and 1.6 mM (each) for two different amplification times (3 h and 14 h).

The experiment in Supplementary Figure 7 simulates a last round RCA reaction. For this, 3 synthetic templates (67, 77 and 87 nt) with a randomized target sequence were circularized and aliquots were amplified in parallel with different dNTP concentration. After the indicated times, the reactions were heat inactivated, a nicking primer was added and the RCA product was digested to monomers with Nt.BspQI. Only one of the two nicking enzyme

was used to generate an easy to analyze digestion pattern where only template monomers and multimers, but no fragments containing unequal numbers of target and intervening sequences are produced. The digestion products were analyzed by denaturing PAGE and the intensities of the gel images were extracted by imageJ. In the left diagram, the normalized total mass of RCA products (monomers and all multimers) was analyzed. The right diagram shows the normalized mass of digested monomers alone.

The left diagram reveals that higher dNTP concentrations yield more RCA product, therefore no inhibition of the polymerase seems to take place here. However, the normalized mass of digested monomers reach a maximum at 0.4 mM. Therefore, a final concentration of 0.4 mM may be more optimal for the production of oligonucleotides than the ~0.9 mM used in the protocol. The differences between the two conditions are however only a few percent. The data further suggests, that shortening the RCA reaction time to 3 hours does not drastically decrease the yield.
